# Discontinuation of Cholinesterase Inhibitors Following Initiation of Memantine and Admission to Long-Term Care Among Older Adults

**DOI:** 10.1001/jamanetworkopen.2024.45878

**Published:** 2024-11-19

**Authors:** Yu-Chien Lee, Sandra M. Shi, Stephanie M. Sison, Chan Mi Park, Gahee Oh, Sohyun Jeong, Ellen P. McCarthy, Dae Hyun Kim

**Affiliations:** 1 Department of Family Medicine, Chang Gung Memorial Hospital, Linkou, Taoyuan, Taiwan; 2 Marcus Institute for Aging Research, Hebrew SeniorLife, Boston, Massachusetts; 3 Department of Medicine, University of Massachusetts Chan Medical School, Worcester; 4 Department of Pharmacy Practice, Massachusetts College of Pharmacy and Health Science, Boston; 5 Division of Gerontology, Department of Medicine, Beth Israel Deaconess Medical Center, Boston, Massachusetts

## Abstract

**Importance:**

Discontinuing cholinesterase inhibitors when initiating memantine in patients with dementia may be reasonable to reduce treatment burden, costs, and the risk of adverse drug events.

**Objective:**

To assess the association of cholinesterase inhibitor discontinuation on long-term care institutionalization among older adults with dementia who initiate memantine.

**Design, Setting, and Participants:**

This retrospective propensity score-matched cohort study used Medicare claims data from January 2014 to December 2019. Participants included fee-for-service Medicare beneficiaries with dementia. Data were analyzed from September 2021 to August 2024.

**Exposures:**

Discontinuation vs continuation of cholinesterase inhibitor.

**Main Outcomes and Measures:**

The primary outcome was 1-year long-term care institutionalization-free days. Secondary outcomes include all-cause death and adverse drug events over 1 year. We performed subgroup analyses based on age, sex, dementia type (Alzheimer disease vs other), frailty, and dementia severity (mild vs moderate or severe) based on claims-based algorithms. The primary outcome was analyzed using nonparametric restricted mean survival time analysis.

**Results:**

Among 16 292 beneficiaries who initiated memantine, 1820 (11.2%) discontinued cholinesterase inhibitors. In the propensity score-matched cohort of 3612 beneficiaries, the mean (SD) age was 80.7 (6.7) years, 2261 (62.6%) were female, and 1989 (55.0%) had a diagnosis of Alzheimer disease. Over 1 year, long-term care institutionalization occurred in 51 of 1806 beneficiaries (2.8%) who discontinued cholinesterase inhibitors (3.4 per 100 person-years) and 62 of 1806 beneficiaries (3.4%) who continued (4.1 per 100 person-years). There was no statistically significant difference in the 1-year mean institutionalization-free days between discontinuation and continuation groups (360.6 [95% CI, 359.3 to 362.0] days vs 359.1 [95% CI, 357.5 to 360.6] days; mean difference, 1.5 [95% CI,−0.5 to 3.6] days). The mean difference in the long-term care institutionalization-free days did not differ by age category, sex, dementia type, frailty, or dementia stage. Individuals who discontinued had a lower rate of fall-related injury (0.9 vs 2.0 per 100 person-years; hazard ratio [HR], 0.47 [95% CI, 0.25 to 0.88]). There was no difference between the discontinuation and continuation groups in all-cause death (10.4 vs 11.6 per 100 person-years; HR, 0.89 [95% CI, 0.72 to 1.10]).

**Conclusions and Relevance:**

In this study, discontinuing cholinesterase inhibitors upon memantine initiation was not associated with an increased risk of long-term care institutionalization but with a lower risk of fall-related injury among older adults with dementia. These findings offer valuable insights for clinicians aiming to reduce treatment burden in this population.

## Key Points

**Question:**

Is discontinuing cholinesterase inhibitors upon memantine initiation associated with an increased risk of long-term care institutionalization among community-dwelling older adults with dementia?

**Findings:**

In this comparative effectiveness study of 3612 Medicare beneficiaries with dementia receiving cholinesterase inhibitors who initiated memantine, those who discontinued the cholinesterase inhibitor had similar 1-year mean long-term care institutionalization-free days and all-cause mortality, but had a lower risk of fall-related injury compared with those who continued the cholinesterase inhibitor.

**Meaning:**

These findings suggest that discontinuing cholinesterase inhibitors when initiating memantine in older adults with dementia may be a reasonable approach to reduce treatment burden.

## Teaser

This comparative effectiveness research study assesses the association of cholinesterase inhibitor discontinuation with long-term care institutionalization among older adults with dementia who initiate memantine.

## Introduction

Cholinesterase inhibitors (donepezil, galantamine, and rivastigmine)^1,2^ and memantine^3^ are commonly prescribed for the treatment of Alzheimer disease (AD) and related dementias. These drugs provide modest, short-term improvements in cognition, behavioral, and psychological symptoms of dementia. Randomized clinical trials indicate that cholinesterase inhibitors benefit patients with mild, moderate, and severe AD,^1^ whereas memantine primarily benefits patients with moderate-to-severe AD.^3^ Consequently, it is a common practice to start cholinesterase inhibitor therapy and then add memantine once patients progress to moderate-to-severe dementia. Although the combination therapy may improve cognition and activities of daily living over 3 to 6 months compared with cholinesterase inhibitor monotherapy,^4,5^ the clinical significance of these effects remains uncertain.^5,6^ A few trials comparing the combination therapy and memantine monotherapy found no difference in cognition and activities of daily living.^7,8^ Additionally, the combination therapy increases the overall treatment burden and costs. Cholinesterase inhibitors also carry risks, including syncope and bradycardia, which can lead to pacemaker insertion and hip fractures.^9,10,11^ Considering the uncertain clinically meaningful benefits and the potential risks associated with their use in conjunction with memantine, cholinesterase inhibitors may be a target for deprescribing. Yet, evidence is lacking on the clinical outcomes of discontinuing cholinesterase inhibitors when adding memantine.

The objective of this study was to evaluate the clinical outcomes associated with discontinuing vs continuing cholinesterase inhibitors upon initiating memantine in community-dwelling older adults with dementia. We examined long-term care institutionalization, mortality, and adverse drug events in Medicare claims data. Although discontinuation is not synonymous with deprescribing, our study may provide valuable insights and motivate further research on deprescribing and medication optimization for older adults living with dementia.

## Methods

### Data Source

This comparative effectiveness study was approved by Advarra, which serves as the institutional review board (IRB) for Hebrew SeniorLife in Boston, Massachusetts. The Advarra IRB granted a waiver of informed consent, which is typical for Medicare claims data analysis. We analyzed data from the Master Beneficiary Summary File, chronic conditions segment file, other chronic or potentially disabling conditions segment file, inpatient, outpatient, skilled nursing facility, home health, carrier, durable medical equipment, prescription drug event, and Minimum Data Set (MDS) from a 5% random sample of Medicare fee-for-service beneficiaries from January 2014 to December 2019.

### Study Design, Population, and Exposure

This study follows the International Society for Pharmacoeconomics and Outcomes Research (ISPOR) reporting guideline^12^ for comparative effectiveness research. The study design is depicted in eFigure 1 of Supplement 1. Our target population was community-dwelling Medicare beneficiaries with AD or related dementias who were receiving cholinesterase inhibitor therapy for at least 90 days and initiated memantine (or a donepezil-memantine combination drug). The date on which memantine or the combination drug was newly prescribed was the index date. The treatment strategies—discontinuing or continuing the cholinesterase inhibitor after memantine initiation—were determined during the 15-day period after the index date (exposure-defining period). Cholinesterase inhibitors were deemed discontinued if the last day of medication supply ended before the exposure-defining period. The outcome follow-up began on the sixteenth day from the index date until the occurrence of outcomes or censoring events.

Medicare beneficiaries were eligible if they were 65 years or older and filled a prescription of memantine between July 1, 2014, and June 30, 2019, without prior fills in the preceding 183 days. Excluded were beneficiaries who (1) did not have continuous enrollment in Medicare Part A, B, and D in the prior 183 days; (2) had a long-term care institutionalization prior to initiating memantine or during the exposure-defining period; (3) had no diagnosis codes indicating AD and related dementias defined using the Chronic Condition Data Warehouse algorithm^13^ before memantine initiation; (4) had potential contraindications to cholinesterase inhibitors or memantine (eg, asthma, atrioventricular block, bradycardia, chronic kidney disease stage 5 or end-stage kidney disease, chronic obstructive pulmonary disease, cirrhosis, gastrointestinal hemorrhage, hypertensive urgency or emergency, and status epilepticus) in the prior 183 days; (5) did not use a cholinesterase inhibitor (donepezil, galantamine, or rivastigmine) for at least 90 days before initiating memantine to include individuals on a stable dose of a cholinesterase inhibitor; or (6) was admitted to a skilled nursing facility during the exposure-defining period. The list of codes to define study variables is provided in eTable 1 of Supplement 1.

### Other Measurements

We measured age, sex, and race and ethnicity as reported in the Master Beneficiary Summary File. Race and ethnicity categories included Asian or Pacific Islander, Black, Hispanic, White, and other (including American Indian or Alaska Native, other, or unknown). Race and ethnicity were considered in this study because they are associated with adherence to dementia medications and long-term nursing home admission.

We also measured dual eligibility for Medicare and Medicaid, the social deprivation index (SDI; range, 0 to 100; greater values indicate more area-level social deprivation based on the zip code),^14^ the Kim claims-based frailty index (range: 0 to 1; greater values indicate more severe frailty),^15,16,17,18^ the Gagne comorbidity score,^19^ type of dementia (AD vs other dementia), time since the first occurrence of dementia diagnosis in Medicare (less than 1 year; 1 to 3 years; more than 3 years) as reported in the chronic conditions segment file, chronic and potentially disabling conditions defined by the Chronic Condition Data Warehouse algorithms,^13^ number of medication classes according to the Anatomical Therapeutic Chemical classification system, health care utilization (numbers and days of inpatient stays, skilled nursing facility stays, and home health days), geographic location, and the calendar time of cohort entry. Cumulative days of inpatient stays, skilled nursing facility stays, and home health were assessed. The Kim claims-based frailty index of 0.25 or more has 60% sensitivity and 86% specificity for identifying the deficit accumulation frailty and 62% sensitivity and 78% specificity for the Fried frailty phenotype.^20^ Moreover, the Kim claims-based frailty index or 0.28 or more has 77% sensitivity and 63% specificity for identifying moderate-to-severe dementia by the Functional Assessment Staging Test stage 5 to 7.^21^ All covariates were measured within 183 days prior to and on the index date.

### Outcomes and Follow-Up

The primary outcome was long-term care institutionalization, which was defined as having more than 100 cumulative days in a nursing facility. The onset of long-term care institutionalization was day 101 in a nursing facility, as obtained from MDS, a federally mandated assessment for nursing home residents. Alternatively, long-term care institutionalization was defined using the Residential History File, which tracks beneficiaries’ health care locations based on Medicare claims and MDS data.^22^ This definition was used in a sensitivity analysis. Secondary outcomes include death and potentially treatment-related adverse events. Vital status was obtained from the Master Beneficiary Summary File. Treatment-related adverse events were defined using diagnosis codes for atrioventricular block, bradycardia, or syncope in the primary position on inpatient claims and diagnosis codes for fall-related injury^23,24^ in any position on inpatient, outpatient, or carrier claims. The claims-based algorithm for fall-related injury had a positive predictive value of 98.8%.^24^ The list of codes is provided in eTable 1 of Supplement 1. Analogous to the intention-to-treat analysis, beneficiaries were followed from the day after the exposure-defining period until the earliest occurrence of (1) outcome of interest; (2) death (in the analysis of outcomes other than death); (3) disenrollment from Medicare Part A, B, and D; (4) end of the study period (December 31, 2019); or (5) 365 days from the start of outcome follow-up.

### Statistical Analysis

To reduce confounding due to the differences in baseline characteristics between the treatment strategies, we used 1:1 nearest neighbor propensity score matching. The propensity score for discontinuing cholinesterase inhibitors was estimated from logistic regression, including the above-listed covariates with at least 1% prevalence in the overall population. A standardized mean difference less than 0.1 was considered adequate. In the primary outcome analysis, we compared the 1-year long-term care institutionalization-free days (ie, the number of days from the day after the exposure defining period until the onset of long-term care episode within one year) between the treatment groups using restricted mean survival time (RMST) analysis,^25,26,27,28,29^ treating death as a censoring event. The mean differences (95% CI) in 1-year RMST can be interpreted as the difference in long-term care institutionalization-free days between the treatment strategies over 1 year. We also compared the Kaplan-Meier long-term care institutionalization-free probabilities using log-rank test. We estimated the RMST difference by prespecified subgroups defined by age (younger than 80 years or 80 years or older), sex, dementia type (AD vs other dementias), frailty (frailty vs no frailty), and dementia stage (mild vs moderate-to-severe dementia). Propensity score estimation and matching were performed within each subgroup, and the heterogeneity of treatment effect between subgroups was tested using the *Z* test. In the analysis of secondary outcomes, we estimated the incidence rates, hazard ratios (HRs), and 95% CIs for death and treatment-related adverse events. For nonmortality secondary outcomes, death was treated as a competing risk and the Fine and Grey competing risk regression was used to estimate the subdistribution HRs. To test the robustness of our primary outcome analysis, we conducted the following sensitivity analyses: (1) extending the exposure-defining period from 15 days to 30 days to assess cholinesterase inhibitor discontinuation; (2) using an alternative definition of long-term care institutionalization based on the Residential History File^22^; (3) using long-term care institutionalization or death as a composite outcome; (4) performing an as-treated analysis, in which beneficiaries were censored when they were no longer adherent to the initial treatment strategy; (5) adjusting for nonadherence using the inverse probability of censoring weighting^30^; and (6) using the alternative Bynum algorithm for dementia identification.^31,32^ Analyses were performed in Stata version 18.0 (StataCorp). A 2-sided *P* < .05 was considered statistically significant. Data were analyzed from September 2021 to August 2024.

## Results

### Characteristics of Study Population

**Figure 1.**
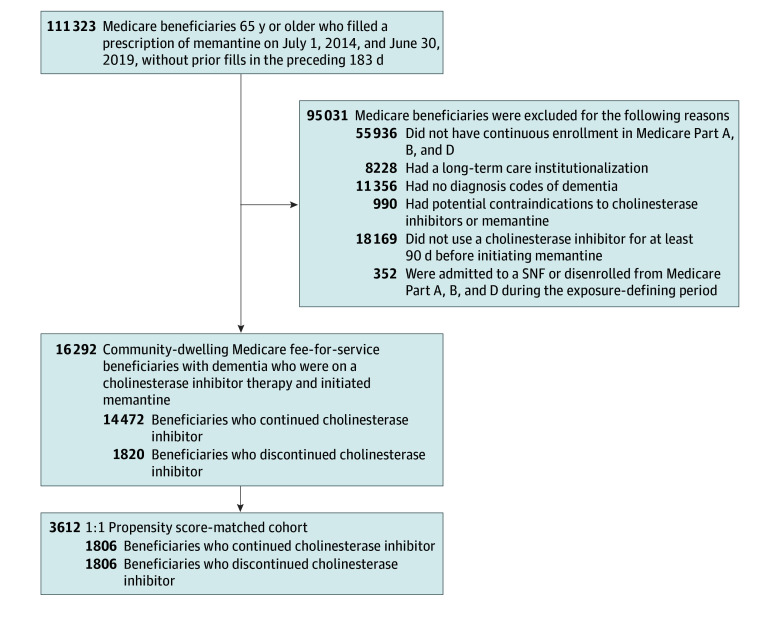
Selection of Study Population SNF indicates skilled nursing facility.

Of 111 323 Medicare beneficiaries who were 65 years or older and filled a memantine prescription between July 1, 2014, and June 30, 2019, we identified 16 292 community-dwelling fee-for-service beneficiaries who initiated memantine while receiving cholinesterase inhibitor therapy in the prior 90 days (Figure 1). Of these, 1820 (11.2%) discontinued cholinesterase inhibitors.

Before matching, the groups were similar in terms of the mean (SD) age (discontinuers vs continuers: 80.6 [6.8] vs 80.8 [6.8] years); the proportions of female (1145 of 1820 [62.9%] vs 9131 of 14 472 [63.1%]); Asian beneficiaries (55 [3.0%] vs 369 [2.5%]), Black beneficiaries (124 [6.8%] vs 941 [6.5%]), Hispanic beneficiaries (122 [6.7%] vs 815 [5.6%]); White beneficiaries (1489 of 1820 [81.8%] vs 12 159 of 14 472 [84.0%]); dual eligible (255 [14.0%] vs 188 [14.3%]); and mean (SD) SDI (46.2 [27.9] vs 45.3 [27.6]) (Table 1 and eTable 2 in Supplement 1). Compared with those who continued cholinesterase inhibitors, the beneficiaries who discontinued were less likely to have the diagnosis codes for AD (discontinued vs continued: 1010 of 1820 [55.5%] vs 8830 [61.0%]) and a higher percentage of people who had the diagnosis of dementia for less than 1 year (less than 1 year since diagnosis: 684 [37.6%] vs 4315 [29.8%]). Although most characteristics were comparable between the groups, the discontinuers had a greater mean (SD) number of inpatient stays (discontinuers vs continuers: 0.26 [0.6] vs 0.20 [0.6]) and skilled nursing facility stays (0.10 [0.4] vs 0.05 [0.3]) in the prior 183 days.

**Table 1.** Characteristics of Medicare Beneficiaries With Dementia Who Discontinued or Continued Cholinesterase Inhibitors Upon Initiating Memantine

Characteristics	Patients, No. (%)					
	Before propensity score matching			After propensity score matching		
	Discontinuers (n = 1820)	Continuers (n = 14 472)	SMD	Discontinuers (n = 1806)	Continuers (n = 1806)	SMD
Age, mean (SD), y	80.6 (6.8)	80.8 (6.8)	−0.03	80.6 (6.8)	80.7 (6.7)	−0.01
Sex						
Female	1145 (62.9)	9131 (63.1)	−0.004	1133 (62.7)	1128 (62.5)	−0.006
Male	675 (37.1)	5341 (36.9)		673 (37.3)	678 (37.5)	
Race and ethnicity						
Asian	55 (3.0)	369 (2.5)	−0.07	55 (3.0)	49 (2.7)	−0.07
Black	124 (6.8)	941 (6.5)		120 (6.6)	94 (5.2)	
Hispanic	122 (6.7)	815 (5.6)		116 (6.4)	117 (6.5)	
White	1489 (81.8)	12 159 (84.0)		1485 (82.2)	1510 (83.6)	
Other^a^	30 (1.6)	188 (1.3)		30 (1.7)	36 (2.0)	
Dual eligibility^b^	255 (14.0)	2068 (14.3)	−0.008	252 (14.0)	241 (13.3)	−0.02
Social deprivation index, mean (SD)^c^	46.2 (27.9)	45.3 (27.6)	0.03	46.1 (27.9)	47.0 (27.3)	−0.03
Kim CFI, mean (SD)	0.2 (0.1)	0.2 (0.1)	0.05	0.2 (0.1)	0.2 (0.1)	−0.04
Gagne CCS, mean (SD)	3.4 (2.7)	3.3 (2.6)	0.06	3.4 (2.6)	3.4 (2.7)	0.006
Type of dementia						
Alzheimer disease	1010 (55.5)	8830 (61.0)	−0.11	998 (55.3)	991 (54.9)	−0.008
Other	810 (44.5)	5642 (39.0)		808 (44.7)	815 (45.1)	
Time since dementia diagnosis, y						
<1	684 (37.6)	4315 (29.8)	−0.17	678 (37.5)	682 (37.8)	−0.01
1-3	621 (34.1)	5222 (36.1)		619 (34.3)	609 (33.7)	
>3	515 (28.3)	4935 (34.1)		509 (28.2)	515 (28.5)	
Comorbidities^d^						
Acute myocardial infarction	105 (5.8)	805 (5.6)	−0.009	104 (5.8)	89 (4.9)	−0.04
Anxiety disorder	671 (36.9)	5338 (36.9)	0	664 (36.8)	654 (36.2)	−0.01
Atrial fibrillation	363 (19.9)	2819 (19.5)	−0.01	361 (20.0)	360 (19.9)	−0.001
Bipolar disorder	92 (5.1)	813 (5.6)	−0.03	89 (4.9)	114 (6.3)	−0.06
Chronic kidney disease	706 (38.8)	5537 (38.3)	−0.01	698 (38.6)	696 (38.5)	−0.002
COPD	579 (31.8)	4433 (30.6)	−0.03	575 (31.8)	546 (30.2)	−0.04
Depression	1009 (55.4)	8085 (55.9)	−0.009	999 (55.3)	1025 (56.8)	−0.03
Diabetes	787 (43.2)	6120 (42.3)	−0.02	779 (43.1)	803 (44.5)	−0.03
Heart failure	590 (32.4)	4789 (33.1)	−0.01	582 (32.2)	558 (30.9)	−0.03
Hip fracture	110 (6.0)	921 (6.4)	−0.01	108 (6.0)	102 (5.6)	−0.01
Ischemic heart disease	1093 (60.1)	8672 (59.9)	−0.003	1082 (59.9)	1098 (60.8)	−0.02
Stroke	509 (28.0)	3998 (27.6)	−0.008	502 (27.8)	515 (28.5)	−0.02
Medications, mean (SD)^e^	9.0 (4.7)	9.0 (4.7)	0.005	9.0 (4.7)	9.1 (4.9)	−0.02
Medications						
Antidepressants	860 (47.3)	7394 (51.1)	−0.08	853 (47.2)	879 (48.7)	−0.03
Antiepileptics	341 (18.7)	3010 (20.8)	−0.05	337 (18.7)	363 (20.1)	−0.04
Antipsychotics	235 (12.9)	1935 (13.4)	−0.01	234 (13.0)	231 (12.8)	−0.005
Anxiolytics	260 (14.3)	2287 (15.8)	−0.04	257 (14.2)	231 (12.8)	−0.04
Benzodiazepines	209 (11.5)	1935 (13.4)	−0.06	206 (11.4)	184 (10.2)	−0.04
Hypnotics or sedatives	93 (5.1)	695 (4.8)	−0.01	93 (5.1)	88 (4.9)	−0.01
Opioids	281 (15.4)	2137 (14.8)	−0.01	278 (15.4)	277 (15.3)	−0.002
Health care utilization, mean (SD)						
Inpatient stays	0.3 (0.6)	0.2 (0.6)	0.11	0.3 (0.6)	0.3 (0.6)	−0.02
Skilled nursing facility stays	0.10 (0.4)	0.05 (0.3)	0.13	0.09 (0.4)	0.09 (0.4)	0
Home health days	12.0 (32.1)	12.3 (33.8)	−0.01	12.0 (32.2)	12.3 (32.7)	−0.01

Abbreviations: CCS, combined comorbidity score; CFI, claims-based frailty index; COPD, chronic obstructive pulmonary disease; SMD, standardized mean difference.

^a^ Other race category includes American Indian or Alaska native, other, and unknown.

^b^ Dual eligibility for Medicare and Medicaid.

^c^ SDI indicates the area-level social deprivation based on the zip code level.

^d^ The diagnosis of comorbidities, medications, and health care utilization were assessed during the 183 days before the initiation of memantine.

^e^ Mean number of medication classes was calculated according to the Anatomical Therapeutic Chemical level 3 classification.

The propensity score-matched population included 1806 pairs of cholinesterase inhibitors discontinuers and continuers (Figure 1). After matching, all characteristics were well-balanced, as measured by standardized mean difference less than 0.1 (Table 1; eTable 2 and eFigure 2 in Supplement 1). In the propensity score-matched cohort of 3612 beneficiaries, the mean (SD) age was 80.7 (6.7) years, 2261 (62.6%) were female, and 1989 (55.0%) had a diagnosis of AD. The characteristics of the matched population were similar to those of the discontinuers. 

### Long-Term Care Institutionalization

**Figure 2.**
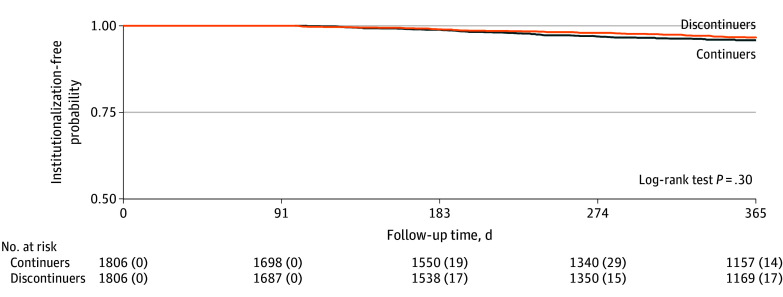
Kaplan-Meier Curves for Long-Term Care Institutionalization Among Medicare Beneficiaries With Dementia Who Discontinued or Continued Cholinesterase Inhibitors Upon Initiating Memantine The No. at risk and the number of the events within each interval (in parentheses) are shown below the graph.

In the propensity score-matched population, 51 of 1806 beneficiaries (2.8%) who discontinued (3.4 per 100 person-years) and 62 of 1806 beneficiaries (3.4%) who continued cholinesterase inhibitors (4.1 per 100 person-years) were admitted to a long-term care institution over a mean (SD) follow-up of 10.2 (3.3) months, with no statistically significant difference between the treatment groups (log-rank test, *P* = .30) (Figure 2). The mean 1-year long-term care institutionalization-free days was 360.6 (95% CI, 359.3 to 362.0) for those who discontinued cholinesterase inhibitors and 359.1 (95% CI, 357.5 to 360.6) days for those who continued, with a mean difference of 1.5 (95% CI, −0.5 to 3.6) days (Table 2). These results indicated that the difference in the long-term care institutionalization-free days could range from 0.5 days (in favor of continuation) to 3.6 days (in favor of discontinuation) over 1 year. The results did not differ by age category, sex, dementia type, frailty, or dementia severity.

**Table 2.** Long-Term Care Institutionalization Among Medicare Beneficiaries With Dementia Who Discontinued or Continued Cholinesterase Inhibitors Upon Initiating Memantine

**Outcome and treatment group**	**Events, No.**	**Follow-up, P-Y**	**Incidence rate, events per 100 P-Y**	**1-y event-free days (95% CI), d**	**Mean difference (95% CI), d**	***P* value for heterogeneity**
**Total population (n = 1806)**						
Discontinuers (n = 1806)	51	1516.5	3.4	360.6 (359.3 to 362.0)	1.5 (−0.5 to 3.6)	NA
Continuers (n = 1806)	62	1516.9	4.1	359.1 (357.5 to 360.6)	1 [Reference]	
**Subgroup by age category**						
Age <80 y						.06
Discontinuers (n = 819)	24	699.7	3.4	360.3 (358.1 to 362.3)	−0.1 (−3.0 to 2.9)	
Continuers (n = 819)	23	696.2	3.3	360.3 (358.3 to 362.4)	1 [Reference]	
Age ≥80 y						
Discontinuers (n = 925)	26	766.5	3.4	360.9 (359.1 to 362.7)	4.1 (0.9 to 7.3)	
Continuers (n = 925)	43	766.4	5.6	356.8 (354.1 to 359.5)	1 [Reference]	
**Subgroup by sex**						
Female						.72
Discontinuers (n = 1104)	38	932.6	4.1	359.6 (357.6 to 361.5)	0.2 (−2.5 to 3.0)	
Continuers (n = 1104)	39	953.8	4.1	359.3 (357.4 to 361.3)	1 [Reference]	
Male						
Discontinuers (n = 640)	12	533.8	2.2	362.4 (360.6 to 364.1)	0.9 (−1.9 to 3.6)	
Continuers (n = 640)	12	537.0	2.2	361.5 (359.4 to 363.6)	1 [Reference]	
**Subgroup by dementia type**						
Alzheimer disease						.53
Discontinuers (n = 964)	32	794.1	4.0	359.5 (357.3 to 361.6)	1.5 (−1.7 to 4.6)	
Continuers (n = 964)	40	812.4	4.9	358.0 (355.7 to 360.3)	1 [Reference]	
Other dementia						
Discontinuers (n = 779)	18	671.6	2.7	361.9 (360.2 to 363.6)	2.9 (−0.1 to 6.0)	
Continuers (n = 779)	23	657.6	3.5	359.0 (356.4 to 361.5)	1 [Reference]	
**Subgroup by frailty** ^a^						
Nonfrail						.31
Discontinuers (n = 1056)	20	902.7	2.2	362.4 (361.1 to 363.8)	3.8 (1.3 to 6.3)	
Continuers (n = 1056)	37	913.8	4.0	358.7 (356.5 to 360.8)	1 [Reference]	
Frail						
Discontinuers (n = 684)	29	560.5	5.2	358.0 (355.1 to 360.8)	1.3 (−2.9 to 5.4)	
Continuers (n = 684)	33	561.7	5.9	356.7 (353.7 to 359.7)	1 [Reference]	
**Subgroup by dementia severity** ^b^						
Mild dementia						.57
Discontinuers (n = 1315)	26	1118.9	2.3	362.1 (360.8 to 363.4)	1.2 (−0.8 to 3.1)	
Continuers (n = 1315)	33	1132.0	2.9	360.9 (359.4 to 362.4)	1 [Reference]	
Moderate-to-severe dementia						
Discontinuers (n = 427)	23	347.2	6.6	356.4 (352.6 to 360.3)	−0.5 (−6.0 to 5.1)	
Continuers (n = 427)	18	348.3	5.2	356.9 (352.9 to 360.8)	1 [Reference]	

Abbreviation: P-Y, person-years.

^a^ The Kim claims-based frailty index of 0.25 or more has 60% sensitivity and 86% specificity for identifying the deficit accumulation frailty and 62% sensitivity and 78% specificity for the Fried frailty phenotype.^19^

^b^ In older adults with dementia, the Kim claims-based frailty index of 0.28 or more has 77% sensitivity and 63% specificity for identifying moderate-to-severe dementia by the Functional Assessment Staging Test stage 5 to 7.^20^

### Death and Potentially Treatment-Related Adverse Events

In the propensity score-matched population, the mortality rate was similar between the treatment groups (discontinuers vs continuers: 10.4 vs 11.6 per 100 person-years; HR, 0.89 [95% CI, 0.72 to 1.10]) (Table 3). Compared with beneficiaries who continued cholinesterase inhibitors, those who discontinued had a lower rate of fall-related injury (0.9 vs 2.0 per 100 person-years; HR, 0.47 [95% CI, 0.25 to 0.88]). Atrioventricular block, bradycardia, and syncope were rare events in this population.

**Table 3.** Mortality and Adverse Events Among Medicare Beneficiaries With Dementia Who Discontinued or Continued Cholinesterase Inhibitors Upon Initiating Memantine

Treatment strategy	Events, No.	Follow-up, person-years	Incidence rate, events per 100 person-years	HR (95% CI)
**Mortality**				
Discontinuers	159	1531.5	10.4	0.89 (0.72-1.10)
Continuers	179	1538.0	11.6	1 [Reference]
**Fall-related injury**				
Discontinuers	14	1522.8	0.9	0.47 (0.25-0.88)^a^
Continuers	30	1523.0	2.0	1 [Reference]
**AV block, bradycardia, or syncope**				
Discontinuers	NR^b^	1529.8	NR^b^	0.88 (0.32-2.43)^a^
Continuers	NR^b^	1533.8	NR^b^	1 [Reference]

Abbreviations: AV, atrioventricular; HR, hazard ratio; NR, not reported.

^a^ Subdistribution hazard ratios were estimated from Fine and Grey competing risk model.

^b^ The cell size less than 11 events is suppressed in compliance to the Centers for Medicare & Medicaid Services policy.

### Sensitivity Analyses

The results remained consistent with the primary analysis when we used the 30-day exposure-defining period, an alternative definition of long-term care institutionalization, a composite of long-term care institutionalization or death, an as-treated analysis, and the Bynum algorithm for identifying dementia. These data can be found in eTable 3 in Supplement 1.

## Discussion

In Medicare beneficiaries with AD or related dementias who initiated memantine, we found no clinically significant difference in the long-term care institutionalization-free days between beneficiaries who discontinued cholinesterase inhibitors and those who continued them. The rate of fall-related injury was lower in those who discontinued cholinesterase inhibitors. Our results supported the consideration of deprescribing cholinesterase inhibitors when memantine was initiated, thereby reducing the treatment burden and possibly adverse events associated with treatment.

A few studies have examined whether cholinesterase inhibitors and memantine can delay long-term care institutionalization. In a randomized clinical trial^33^ of 565 patients with mild-to-moderate AD, donepezil did not delay long-term care institutionalization over 2 years. In a randomized clinical trial^34^ of 295 patients with moderate-to-severe AD who were receiving a stable cholinesterase inhibitor therapy, discontinuation of donepezil was not associated with a difference in long-term care institutionalization at 3 years. These trials, which were conducted in the UK, may not be generalizable to the US Medicare beneficiaries due to the differences in health care systems and practices affecting long-term care institutionalization. In the US, an observational study^9^ using Medicare fee-for-service claims from 73 475 beneficiaries who were newly diagnosed with AD found no difference in skilled nursing facility admissions among cholinesterase inhibitor initiators, memantine initiators, and combination therapy initiators. However, the authors neither distinguished between long-term care institutionalization and short-term skilled nursing facility stays, nor did they consider the different stages of dementia in which these treatments are prescribed.

Our study offered evidence for the comparative effectiveness of discontinuing vs continuing cholinesterase inhibitors upon initiating memantine in a routine care population. Long-term care institutionalization is a patient-centered outcome that is associated with cognitive ability, functional status, and neuropsychiatric symptoms,^35^ as well as quality of life^36^ among community-dwelling older adults with dementia. Our primary outcome was analyzed using nonparametric RMST analysis, which did not require the proportional hazards assumption of Cox regression. Compared with HRs, RMST difference between the treatment strategies could be intuitively interpreted as the gain or loss in long-term care institutionalization-free days, which was useful in determining the clinical significance of the results.^25,26,27,28,29^ In our primary analysis, the mean difference of 1.5 (95% CI, −0.5 to 3.6) days showed that the discontinuation was associated with a delay in long-term care institutionalization, on average, by 1.5 days over 1 year compared with the continuation strategy. The mean difference could range from 0.5 days (in favor of continuation) to 3.6 days (in favor of discontinuation). In our study, long-term care institutionalization occurred in low frequency within 1 year. Therefore, our results could not assess longer term benefits and harms of cholinesterase inhibitor discontinuation beyond 1 year. The modest mean difference in RMST in the subgroup aged 80 years or older (4.1 [95% CI, 0.9 to 7.3] days) and the nonfrail subgroup (3.8 [95% CI, 1.3 to 6.3] days) suggested that a clinically meaningful delay in long-term care institutionalization is unlikely.

With regards to adverse events, we observed little difference in mortality between the treatment strategies, similar to the findings of the Donepezil and Memantine in Moderate to Severe Alzheimer Disease trial.^34^ Although the lower rate of fall-related injury among those who discontinued cholinesterase inhibitors was not statistically significant in our study, our finding aligned with previous research that reported a reduction in falls and fractures associated with cholinesterase inhibitor discontinuation in nursing home residents with severe dementia.^37^ Furthermore, while several studies found increased risks of bradycardia and syncope associated with the initiation of cholinesterase inhibitors,^9,10,11^ such events were rare in our study population, which consisted of patients who had previously tolerated cholinesterase inhibitors before starting memantine.

### Limitations

The findings of our study should be interpreted with consideration of the limitations associated with the use of Medicare claims, which were not generated for research purposes. These limitations include measurement errors, misclassification of drug exposure and outcomes, and confounding. Measurements of dementia type, time since diagnosis, and stage of dementia from claims data may be inaccurate. Particularly, we were unable to determine whether cholinesterase inhibitors were discontinued due to lack of efficacy, patient or caregiver preference, or adverse effects. The decision and timing of long-term care institutionalization are affected by several factors that are unmeasured in our data, such as the severity of cognitive impairment, functional impairment, behavioral symptoms, caregiver burnout, social support, and financial status. Lack of clinical and contextual details may lead to residual and unmeasured confounding. In addition, adverse events might have been undercoded in claims data, resulting in low incidence rates. Our study was not adequately powered to detect clinically meaningful differences in death, atrioventricular block, bradycardia, and syncope. Lastly, our results may not generalize to patients who are not enrolled in Medicare fee-for-service plan or those who initiate memantine without concurrent cholinesterase inhibitor therapy. Since our follow-up was 1 year, we were unable to assess the longer-term benefits and harms of cholinesterase inhibitor discontinuation. For certain individuals, pharmacological therapy may help ameliorate disturbing behavioral symptoms, thereby allowing them stay home longer. Our analyses could not find any evidence for the heterogeneity of treatment effect by the subgroups examined.

## Conclusions

In older adults with dementia who received cholinesterase inhibitor therapy and initiate memantine, discontinuing the cholinesterase inhibitor was not associated with long-term care institutionalization, yet with a lower risk of fall-related injury. Given the risks associated with polypharmacy and the growing evidence questioning the patient-centered benefits of the combination therapy, our findings suggest valuable guidance for clinicians in reducing the treatment burden for older adults with dementia.

## Notes

Supplement 1. **eTable 1.** List of Codes to Define Study Variables **eTable 2.** Characteristics of Medicare Beneficiaries With Dementia Who Discontinued or Continued Cholinesterase Inhibitors Upon Initiating Memantine (Complete List of Covariates) **eTable 3. **Sensitivity Analyses **eFigure 1.** Schematic of the Study Design **eFigure 2.** Propensity Score Distribution Before and After 1:1 Propensity Score Matching

Supplement 2.
Data Sharing Statement
