# Supplementary material for: Discontinuation of Cholinesterase Inhibitors Following Initiation of Memantine and Admission to Long-Term Care Among Older Adults
Source: JAMA Netw Open. 2024 Nov 19;7(11):e2445878. doi: 10.1001/jamanetworkopen.2024.45878 (PMC11577138; doi:10.1001/jamanetworkopen.2024.45878)
Supplement: Supplement 1. — eTable 1. List of Codes to Define Study Variables eTable 2. Characteristics of Medicare Beneficiaries With Dementia Who Discontinued or Continued Cholinesterase Inhibitors Upon Initiating Memantine (Complete List of Covariates) eTable 3. Sensitivity Analyses eFigure 1. Schematic of the Study Design eFigure 2. Propensity Score Distribution Before and After 1:1 Propensity Score Matching [file jamanetwopen-e2445878-s001.pdf]

Supplemental Online Content

Lee Y-C, Shi SM, Sison SM, et al. Discontinuation of cholinesterase inhibitors following initiation of memantine and admission to long-term care among older adults. *JAMA Netw Open*. 2024;7(11):e2445878. doi:10.1001/jamanetworkopen.2024.45878

eTable 1. List of Codes to Define Study Variables
eTable 2. Characteristics of Medicare Beneficiaries With Dementia Who Discontinued or Continued Cholinesterase Inhibitors Upon Initiating Memantine (Complete List of Covariates)
eTable 3. Sensitivity Analyses
eFigure 1. Schematic of the Study Design
eFigure 2. Propensity Score Distribution Before and After 1:1 Propensity Score Matching

This supplemental material has been provided by the authors to give readers additional information about their work.

© 2024 Lee Y-C et al. *JAMA Network Open.*

eTable 1. List of Codes to Define Study Variables

Diagnosis	Algorithm
Asthma	ICD9: 493.00, 493.01, 493.02, 493.10, 493.11, 493.12, 493.20, 493.21, 493.22, 493.81, 493.82, 493.90, 493.91, 493.92<br>ICD10: J44.0, J44.1, J44.9, J45.20, J45.21, J45.22, J45.30, J45.31, J45.32, J45.40, J45.41, J45.42, J45.50, J45.51, J45.52, J45.901, J45.902, J45.909, J45.990, J45.991, J45.998
Atrioventricular block	ICD9: 427.81, 427.89<br>ICD10: R00.1
Bradycardia	ICD9: 426.0<br>ICD10: I44.2
Chronic kidney disease stage 5 or end-stage renal disease	ICD9: 585.6, V56.0, V56.1, 38.95, 39.27, 39.95<br>ICD10: I12.0, N18.6, Z49.0, Z49.01, Z49.02, Z94.0, Z99.2<br>CPT4: 36145, 36800, 36832, 36833, 90935, 93990<br>Entitlement for Medicare = ESRD from CMS Form 2728
COPD	ICD9: 490, 491.0, 491.1, 491.8, 491.9, 492.0, 492.8, 491.20, 491.21, 491.22, 494.0, 494.1, 496<br>ICD10: J40, J41.0, J41.1, J41.8, J42, J43.0, J43.1, J43.2, J43.8, J43.9, J44.0, J44.1, J44.9, J47.0, J47.1, J47.9
Cirrhosis	ICD9: 571.2, 571.5<br>ICD10: K70.30, K70.31, K74.60, K74.69, K74.3, K74.4, K74.5
Dementia	ICD9: 331.0, 331.11, 331.19, 331.2, 331.7, 290.0, 290.10, 290.11, 290.12, 290.13, 290.20, 290.21, 290.3, 290.40, 290.41, 290.42, 290.43, 294.0, 294.10, 294.11, 294.20, 294.21, 294.8, 797<br>ICD10: F01.50, F01.51, F02.80, F02.81, F03.90, F03.91, F04, G13.2, G13.8, F05, F06.1, F06.8, G30.0, G30.1, G30.8, G30.9, G31.1, G31.2, G31.01, G31.09, G91.4, G94, R41.81, R54
Fall-related injury	ICD9: E880, E881, E884, E885, and E888<br>ICD10: W01.0XXA, W01.10XA, W01.110A, W01.118A, W01.119A, W01.190A, W01.198A, W05.0XXA, W05.1XXA, W05.2XXA, W06.XXXA, W07.XXXA, W08.XXXA, W10.0XXA, W10.1XXA, W10.2XXA, W10.8XXA, W10.9XXA, W17.81XA, W17.89XA, W18.00XA, W18.02XA, W18.09XA, W18.12XA, W18.2XXA, W18.39XA, W18.49XA, W19.XXXA
Gastrointestinal hemorrhage	ICD9: 456.0, 530.7, 531.00, 531.01, 531.20, 531.21, 531.40, 531.41, 531.60, 531.61, 532.00, 532.01, 532.20, 532.21, 532.40, 532.41, 532.60, 532.61, 533.00, 533.01, 533.20, 533.21, 533.40, 533.41, 533.60, 533.61, 534.00, 534.01, 534.20, 534.21, 534.40, 534.41, 537.83, 578.0, 45.11, 45.13, 45.14, 45.16, 44.43<br>ICD-10: K25.0, K25.1, K25.2, K26.0, K26.1, K26.2, K27.0, K27.1, K27.2, K28.0, K28.1, K28.2, K29.0, K92.0, K92.1, K92.2
Hypertension urgency or emergency	ICD9: 401.0<br>ICD10: I16.0, I16.1, I16.9
Status epilepticus	ICD9: 345.2, 345.3<br>ICD10: G41.X
Syncope	ICD9: 708.2<br>ICD10: R55

© 2024 Lee Y-C et al. *JAMA Network Open.*

eTable 2. Characteristics of Medicare Beneficiaries with Dementia Who Discontinued or Continued Cholinesterase Inhibitors Upon Initiating Memantine (Complete List of Characteristics)

Characteristics	Before PS Matching			After PS Matching		
	Discontinuers (n=1820)	Continuers (n=14472)	SMD	Discontinuers (n=1806)	Continuers (n=1806)	SMD
Age, years, mean (SD)	80.6 (6.8)	80.8 (6.8)	0.027	80.6 (6.8)	80.7 (6.7)	0.013
Female, n (%)	1145 (62.9)	9131 (63.1)	0.004	1133 (62.7)	1128 (62.5)	0.006
Race and ethnicity, n (%)			0.065			0.069
Asian	55 (3.0)	369 (2.5)		55 (3.0)	49 (2.7)	
Black	124 (6.8)	941 (6.5)		120 (6.6)	94 (5.2)	
Hispanic	122 (6.7)	815 (5.6)		116 (6.4)	117 (6.5)	
White	1489 (81.8)	12159 (84.0)		1485 (82.2)	1510 (83.6)	
Other[a]	30 (1.6)	188 (1.3)		30 (1.7)	36 (2.0)	
Dual eligibility[b], n (%)	255 (14.0)	2068 (14.3)	0.008	252 (14.0)	241 (13.3)	0.018
SDI[c], mean (SD)	46.2 (27.9)	45.3 (27.6)	-0.030	46.1 (27.9)	47.0 (27.3)	0.030
Kim CFI, mean (SD)	0.24 (0.1)	0.24 (0.1)	-0.050	0.24 (0.1)	0.24 (0.1)	0.037
Gagne CCS, mean (SD)	3.4 (2.7)	3.3 (2.6)	-0.057	3.4 (2.6)	3.4 (2.7)	-0.006
Type of dementia, n (%)			0.112			0.008
Alzheimer's disease	1010 (55.5)	8830 (61.0)		998 (55.3)	991 (54.9)	
Other	810 (44.5)	5642 (39.0)		808 (44.7)	815 (45.1)	
Time since dementia diagnosis, n (%)			0.173			0.012
<1 year	684 (37.6)	4315 (29.8)		678 (37.5)	682 (37.8)	
1-3 years	621 (34.1)	5222 (36.1)		619 (34.3)	609 (33.7)	
>3 years	515 (28.3)	4935 (34.1)		509 (28.2)	515 (28.5)	
Comorbidities, n (%)						
Acute MI	105 (5.8)	805 (5.6)	0.009	104 (5.8)	89 (4.9)	0.037
Anxiety disorder	671 (36.9)	5338 (36.9)	0.000	664 (36.8)	654 (36.2)	0.012
Arthritis	1307 (71.8)	10550 (72.9)	0.024	1296 (71.8)	1304(72.2)	0.010
Atrial fibrillation	363 (19.9)	2819 (19.5)	0.012	361 (20.0)	360 (19.9)	0.001
Anemia	1304 (71.6)	10281 (71.0)	0.013	1294 (71.7)	1274 (70.5)	0.024
Asthma	312 (17.1)	2302 (15.9)	0.033	309 (17.1)	286 (15.8)	0.034
Alcohol use disorder	67 (3.7)	555 (3.8)	0.008	66 (3.7)	57 (3.2)	0.027
ADHD and other conduct disorders	25 (1.4)	201 (1.4)	0.001	25 (1.4)	21 (1.2)	0.020
Bipolar disorder	92 (5.1)	813 (5.6)	0.025	89 (4.9)	114 (6.3)	0.060
Brain Injury	66 (3.6)	536 (3.7)	0.004	63 (3.5)	61 (3.4)	0.006
BPH	450 (24.7)	3492 (24.1)	0.014	448 (24.8)	440 (24.4)	0.010
Cataract	1530 (84.1)	12083 (83.5)	0.016	1521 (84.2)	1520 (84.2)	0.002
CKD	706 (38.8)	5537 (38.3)	0.011	698 (38.6)	696 (38.5)	0.002
COPD	579 (31.8)	4433 (30.6)	0.026	575 (31.8)	546 (30.2)	0.035
Cystic Fibrosis	54 (3.0)	364 (2.5)	0.028	51 (2.8)	58 (3.2)	0.023
Cancer, breast	139 (7.6)	1108 (7.7)	0.001	139 (7.7)	147 (8.1)	0.016
Cancer, colon	69 (3.8)	489 (3.4)	0.022	69 (3.8)	62 (3.4)	0.021
Cancer, prostate	142 (7.8)	1073 (7.4)	0.015	141 (7.8)	146 (8.1)	0.010

© 2024 Lee Y-C et al. *JAMA Network Open.*

Characteristics	Before PS Matching			After PS Matching		
	Discontinuers (n=1820)	Continuers (n=14472)	SMD	Discontinuers (n=1806)	Continuers (n=1806)	SMD
Cancer, lung	25 (1.4)	205 (1.4)	0.004	25 (1.4)	26 (1.4)	0.005
Cancer, endometrium	11 (0.6)	172 (1.2)	0.062	NR[e]	15 (0.8)	0.033
Depression	1009 (55.4)	8085 (55.9)	0.009	999 (55.3)	1025 (56.8)	0.029
Diabetes	787 (43.2)	6120 (42.3)	0.019	779 (43.1)	803 (44.5)	0.027
Drug use	64 (3.5)	575 (4.0)	0.024	64 (3.5)	62 (3.4)	0.006
Epilepsy	95 (5.2)	774 (5.3)	0.006	91 (5.0)	84 (4.7)	0.018
Fibromyalgia	687 (37.7)	5004 (34.6)	0.066	679 (37.6)	703 (38.9)	0.027
Glaucoma	553 (30.4)	4405 (30.4)	0.001	546 (30.2)	558 (30.9)	0.014
Heart failure	590 (32.4)	4789 (33.1)	0.014	582 (32.2)	558 (30.9)	0.029
Hip fracture	110 (6.0)	921 (6.4)	0.013	108 (6.0)	102 (5.6)	0.014
Hyperlipidemia	1639 (90.1)	13103 (90.5)	0.016	1625 (90.0)	1643 (91.0)	0.034
Hypertension	1646 (90.4)	12918 (89.3)	0.039	1633 (90.4)	1641 (90.9)	0.015
Hypothyroidism	700 (38.5)	5331 (36.8)	0.034	692 (38.3)	696 (38.5)	0.005
Hearing impairment	420 (23.1)	3317 (22.9)	0.004	419 (23.2)	407 (22.5)	0.016
Hepatitis, viral	28 (1.5)	172 (1.2)	0.030	27 (1.5)	18 (1.0)	0.045
Ischemic heart disease	1093 (60.1)	8672 (59.9)	0.003	1082 (59.9)	1098 (60.8)	0.018
Leukemias/lymphomas	47 (2.6)	322 (2.2)	0.023	45 (2.5)	41 (2.3)	0.015
Liver diseases	181 (9.9)	1465 (10.1)	0.006	180 (10)	158 (8.7)	0.042
Mobility impairment	117 (6.4)	850 (5.9)	0.023	114 (6.3)	119 (6.6)	0.011
Migraine	106 (5.8)	902 (6.2)	0.017	104 (5.8)	101 (5.6)	0.007
Major depressive disorder	808 (44.4)	6508 (45.0)	0.012	801 (44.4)	825 (45.7)	0.027
Osteoporosis	639 (35.1)	4977 (34.4)	0.015	634 (35.1)	657 (36.4)	0.027
Obesity	335 (18.4)	2799 (19.3)	0.024	331 (18.3)	327 (18.1)	0.006
Opioid use disorder	37 (2.0)	317 (2.2)	0.011	36 (2.0)	44 (2.4)	0.030
Opioid-related hospitalizations	21 (1.2)	202 (1.4)	0.022	20 (1.1)	26 (1.4)	0.030
Personality disorders	33 (1.8)	338 (2.3)	0.037	33 (1.8)	28 (1.6)	0.021
Peripheral vascular disease	586 (32.2)	4726 (32.7)	0.010	578 (32.0)	600 (33.2)	0.026
Pressure ulcers	180 (9.9)	1608 (11.1)	0.040	177 (9.8)	179 (9.9)	0.004
Stroke	509 (28.0)	3998 (27.6)	0.008	502 (27.8)	515 (28.5)	0.016
Schizophrenia	23 (1.3)	198 (1.4)	0.009	23 (1.3)	29 (1.6)	0.028
Schizophrenia or other psychotic disorders	200 (11.0)	1674 (11.6)	0.018	198 (11.0)	213 (11.8)	0.026
Spinal cord injury	32 (1.8)	207 (1.4)	0.026	30 (1.7)	22 (1.2)	0.037
Tobacco use disorder	170 (9.3)	1457 (10.1)	0.025	170 (9.4)	175 (9.7)	0.009
Visual impairment	44 (2.4)	375 (2.6)	0.011	44 (2.4)	45 (2.5)	0.004
Medications[d], mean (SD)	9.0 (4.7)	9.0 (4.7)	-0.005	9.0 (4.7)	9.1 (4.9)	0.024
Medications, n (%)						
ACEI	482 (26.5)	3959 (27.4)	0.020	480 (26.6)	504 (27.9)	0.030
Antibiotics, penicillin	219 (12.0)	1622 (11.2)	0.026	216 (12.0)	229 (12.7)	0.022

© 2024 Lee Y-C et al. *JAMA Network Open.*

Characteristics	Before PS Matching			After PS Matching		
	Discontinuers (n=1820)	Continuers (n=14472)	SMD	Discontinuers (n=1806)	Continuers (n=1806)	SMD
Antibiotics, beta-lactam	199 (10.9)	1608 (11.1)	0.006	197 (10.9)	209 (11.6)	0.021
Antidepressants	860 (47.3)	7394 (51.1)	0.077	853 (47.2)	879 (48.7)	0.029
Antiepileptics	341 (18.7)	3010 (20.8)	0.052	337 (18.7)	363 (20.1)	0.036
Antipsychotics	235 (12.9)	1935 (13.4)	0.014	234 (13.0)	231 (12.8)	0.005
ARB	403 (22.1)	2983 (20.6)	0.037	398 (22.0)	392 (21.7)	0.008
Anxiolytics	260 (14.3)	2287 (15.8)	0.042	257 (14.2)	231 (12.8)	0.042
Anti-thrombotic drugs	422 (23.2)	3326 (23.0)	0.005	419 (23.2)	429 (23.8)	0.013
Anti-inflammatory drugs	288 (15.8)	2064 (14.3)	0.044	285 (15.8)	286 (15.8)	0.002
Benzodiazepines	209 (11.5)	1935 (13.4)	0.057	206 (11.4)	184 (10.2)	0.039
Beta-blockers	682 (37.5)	5285 (36.5)	0.020	676 (37.4)	660 (36.5)	0.018
CCB	435 (23.9)	3486 (24.1)	0.004	429 (23.8)	424 (23.5)	0.007
Corticosteroids	332 (18.2)	2657 (18.4)	0.003	330 (18.3)	335 (18.5)	0.007
Cough suppressants	168 (9.2)	1490 (10.3)	0.036	168 (9.3)	173 (9.6)	0.009
Diuretics, thiazides	330 (18.1)	2520 (17.4)	0.019	327 (18.1)	323 (17.9)	0.006
Drugs for peptic ulcers and GERD	589 (32.4)	4737 (32.7)	0.008	581 (32.2)	583 (32.3)	0.002
Drugs for BPH	261 (14.3)	1943 (13.4)	0.026	260 (14.4)	258 (14.3)	0.003
Glucose lowering drugs	302 (16.6)	2401 (16.6)	0.000	298 (16.5)	295 (16.3)	0.004
Hypnotics/sedatives	93 (5.1)	695 (4.8)	0.014	93 (5.1)	88 (4.9)	0.013
High-ceiling diuretics	248 (13.6)	2124 (14.7)	0.030	246 (13.6)	240 (13.3)	0.010
Lipid modifying agents	1088 (59.8)	8782 (60.7)	0.018	1079 (59.7)	1134 (62.8)	0.063
Opioids	281 (15.4)	2137 (14.8)	0.019	278 (15.4)	277 (15.3)	0.002
Other analgesics	259 (14.2)	2162 (14.9)	0.020	259 (14.3)	251 (13.9)	0.013
Potassium	194 (10.7)	1818 (12.6)	0.059	191 (10.6)	189 (10.5)	0.004
Quinolones	257 (14.1)	1916 (13.2)	0.026	254 (14.1)	277 (15.3)	0.036
Systemic corticoids	198 (10.9)	1542 (10.7)	0.007	195 (10.8)	210 (11.6)	0.026
Thyroid preparations	442 (24.3)	3590 (24.8)	0.012	440 (24.4)	454 (25.1)	0.018
Urologicals	185 (10.2)	1734 (12.0)	0.059	185 (10.2)	174 (9.6)	0.020
Healthcare utilization, mean (SD)						
Inpatient stays	0.26 (0.6)	0.20 (0.6)	-0.105	0.25 (0.6)	0.27 (0.6)	0.020
Inpatient days	1.7 (5.4)	1.3 (4.5)	-0.091	1.6 (5.2)	1.7 (5.7)	0.024
SNF stays	0.10 (0.4)	0.05 (0.3)	-0.126	0.09 (0.4)	0.09 (0.4)	0.000
SNF days	2.2 (9.6)	1.2 (7.2)	-0.112	2.0 (9.0)	2.1 (9.9)	0.010
Home health days	12.0 (32.1)	12.3 (33.8)	0.010	12.0 (32.2)	12.3 (32.7)	0.010
Geographic region, n (%)			0.082			0.063
New England	84 (4.6)	750 (5.2)		84 (4.7)	75 (4.2)	
Middle Atlantic	247 (13.6)	1864 (12.9)		246 (13.6)	231 (12.8)	
East North Central	274 (15.1)	2328 (16.1)		272 (15.1)	262 (14.5)	
West North Central	110 (6.0)	942 (6.5)		110 (6.1)	121 (6.7)	
South Atlantic	454 (24.9)	3409 (23.6)		452 (25.0)	480 (26.6)	
East South Central	133 (7.3)	1276 (8.8)		131 (7.3)	136 (7.5)	

© 2024 Lee Y-C et al. *JAMA Network Open.*

Characteristics	Before PS Matching			After PS Matching		
	Discontinuers (n=1820)	Continuers (n=14472)	SMD	Discontinuers (n=1806)	Continuers (n=1806)	SMD
West South Central	221 (12.1)	1667 (11.5)		221 (12.2)	214 (11.8)	
Mountain	86 (4.7)	652 (4.5)		86 (4.8)	95 (5.3)	
Pacific or other	211 (11.6)	1584 (10.9)		204 (11.3)	192 (10.6)	
Calendar time, n (%)			0.076			0.065
2014/07-2014/12	200 (11.0)	1397 (9.7)		200 (11.1)	213 (11.8)	
2015/01-2015/06	206 (11.3)	1531 (10.6)		203 (11.2)	227 (12.6)	
2015/07-2015/12	212 (11.6)	1660 (11.5)		210 (11.6)	209 (11.6)	
2016/01-2016/06	215 (11.8)	1769 (12.2)		211 (11.7)	213 (11.8)	
2016/07-2016/12	186 (10.2)	1585 (11.0)		184 (10.2)	170 (9.4)	
2017/01-2017/06	110 (6.0)	946 (6.5)		109 (6.0)	97 (5.4)	
2017/07-2017/12	178 (9.8)	1613 (11.1)		177 (9.8)	179 (9.9)	
2018/01-2018/06	168 (9.2)	1382 (9.5)		167 (9.2)	162 (9.0)	
2018/07-2018/12	174 (9.6)	1294 (8.9)		174 (9.6)	160 (8.9)	
2019/01-2019/06	171 (9.4)	1295 (8.9)		171 (9.5)	176 (9.7)	

Abbreviations: ADHD, attention deficit hyperactivity disorder; ACEI, angiotensin converting enzyme inhibitors; ARB, angiotensin receptor blockers; BPH, benign hypertrophy of prostate; CCB, calcium channel blockers; CCS, combined comorbidity score; CFI, claims-based frailty index; CKD, chronic kidney disease; COPD, chronic obstructive pulmonary disease; GERD, gastroesophageal reflux disorders; MI, myocardial infarction; SD, standard deviation; SDI, social deprivation index; SMD, standardized mean difference; SNF, skilled nursing facility.

Note: The diagnosis of comorbidities, medications, and healthcare utilization are assessed during the covariate assessment period (183 days before index date).

[a] Other race category includes American Indian/Alaska native, other, and unknown.

[b] Dual eligibility for Medicare and Medicaid.

[c] SDI indicates the area-level social deprivation based on the zip code level.

[d] Mean number of medication classes was calculated according to the Anatomical Therapeutic Chemical level 3 classification.

[e] The cell size less than 11 events is suppressed in compliance to the Centers for Medicare & Medicaid Services policy.

eTable 3. Sensitivity Analyses

Treatment Strategy	Events<br>*n*	Follow-Up<br>*py*	Incidence Rate<br>*events per 100 py*	1-Year Event-Free Days (95% CI)<br>*d*	Mean Difference (95% CI)<br>*d*
**Analysis 1: Exposure defining period 30 days**					
Discontinuers (n=2180)	66	1802.4	3.7	359.6 (358.1, 361.0)	0.4 (-1.7, 2.4)
Continuers (n=2180)	69	1822.7	3.8	359.2 (357.7, 360.7)	Reference
**Analysis 2: Alternative definition of long-term care institutionalization based on the Residential History File**					
Discontinuers (n=1805)	113	1493.2	7.6	352.6 (350.1, 355.1)	1.5 (-2.1, 5.2)
Continuers (n=1805)	119	1504.4	7.9	351.0 (348.4, 353.7)	Reference
**Analysis 3: Composite outcome of long-term care institutionalization or death**					
Discontinuers (n=1806)	204	1516.5	13.5	344.0 (340.9, 347.1)	3.0 (-1.5, 7.6)
Continuers (n=1806)	231	1516.9	15.2	341.0 (337.7, 344.2)	Reference
**Analysis 4: As-treated analysis**					
Discontinuers (n=1747)	17	950.7	1.8	362.8 (361.3, 364.3)	1.5 (-1.1, 4.0)
Continuers (n=1747)	16	857.4	1.9	361.4 (359.3, 363.5)	Reference
**Analysis 5: IPCW analysis to adjust for non-adherence**					
Discontinuers (n=2103)	52	3582.9	1.5	364.2 (363.6, 364.7)	0.4 (-0.6, 1.4)
Continuers (n=1955)	26	3231.1	0.8	363.7 (362.9, 364.5)	Reference
**Analysis 6: Definition of dementia based on Bynum algorithm**					
Discontinuers (n=1097)	34	913.6	3.7	360.2 (358.3, 362.0)	0.8 (-2.0, 3.6)
Continuers (n=1097)	34	912.1	3.7	359.4 (357.3, 361.4)	Reference

Abbreviations: CI, confidence interval; d, days; IPCW, inverse probability of censoring weight; py, person-years.

© 2024 Lee Y-C et al. *JAMA Network Open.*

eFigure 1. Schematic of the Study Design

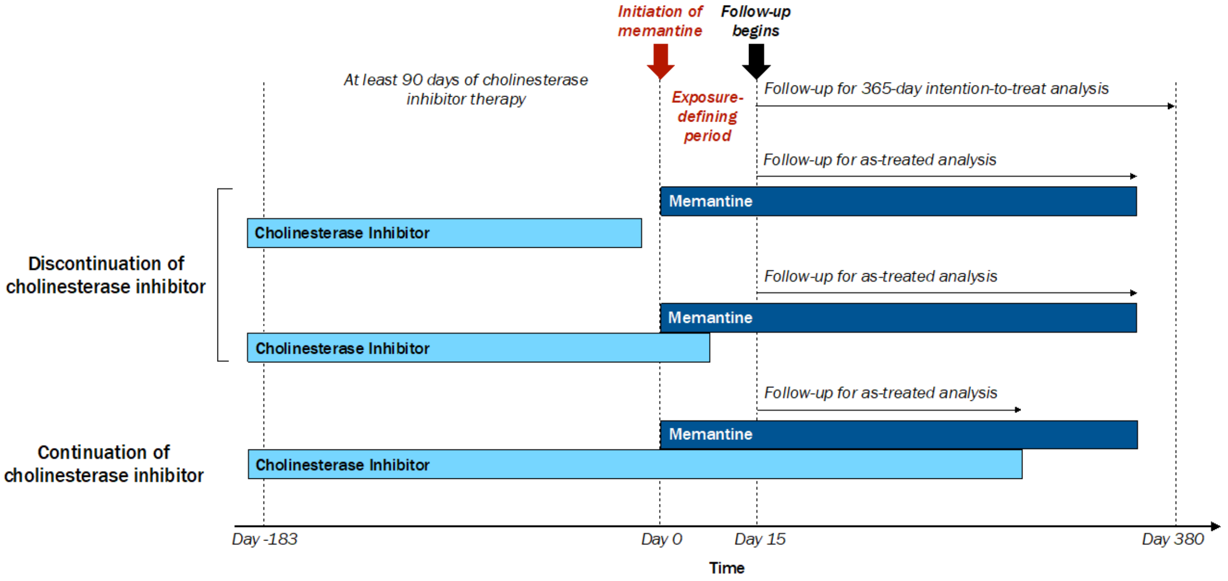

The date on which memantine was newly prescribed was the index date (time *t*). The treatment strategies—discontinuing or continuing the cholinesterase inhibitor after memantine initiation—were determined during the 15-day period after the index date (the exposure-defining period is *t* to *t+15*). Cholinesterase inhibitors were deemed discontinued if no prescription refill occurred within 15 days after the current prescription's supply ended. The outcome follow-up of the intention-to-treat analysis began on the 16th day from the index date (*t+16*) until the earliest occurrence of 1) outcome of interest; 2) death (in the analysis of outcomes other than death); 3) disenrollment from Medicare Part A, B, and D; 4) end of the study period (December 31, 2019); or 5) 365 days from the start of outcome follow-up. In the as-treated analysis, beneficiaries were censored when they were no longer adherent to the initial treatment strategy.

© 2024 Lee Y-C et al. *JAMA Network Open.*

eFigure 2. Propensity Score Distribution Before and After 1:1 Propensity Score Matching

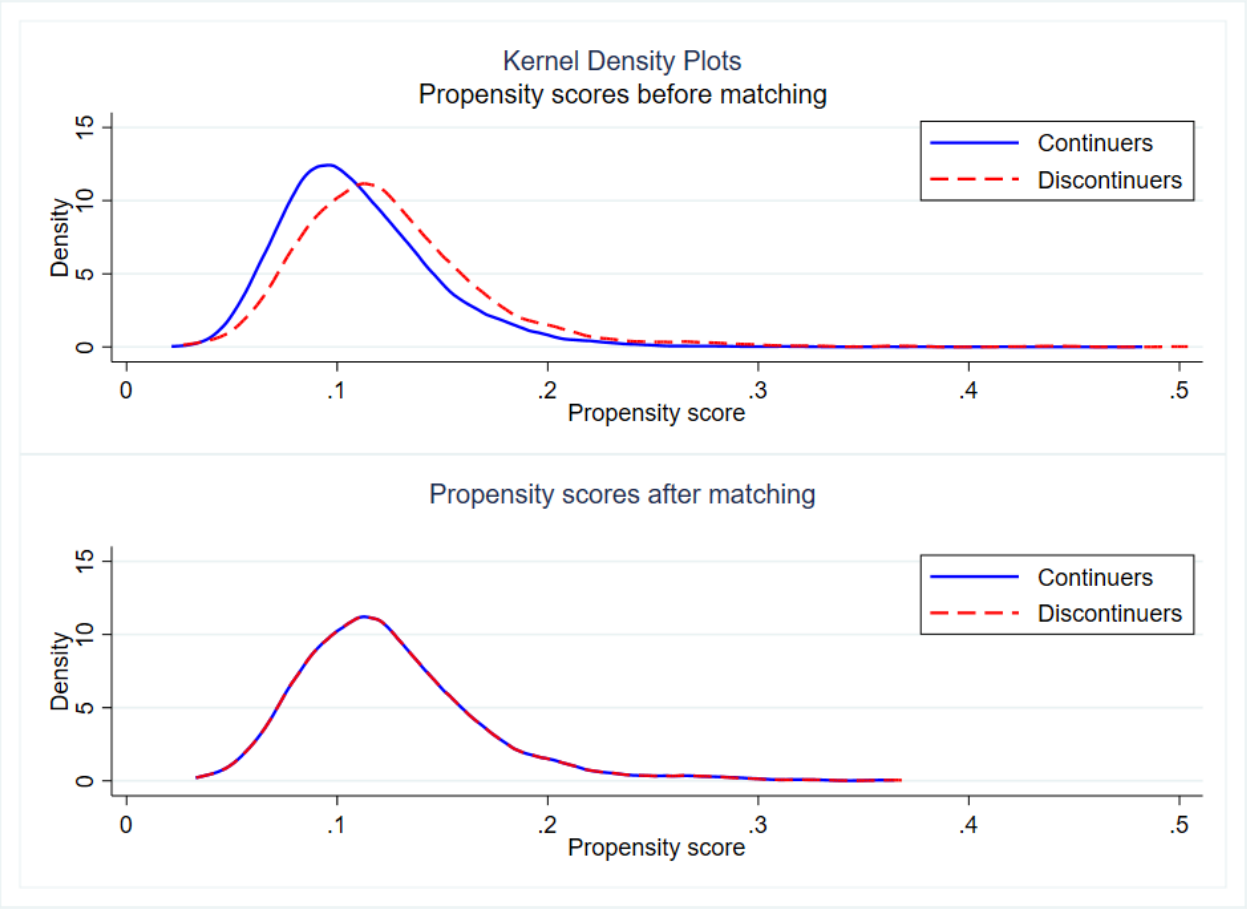

© 2024 Lee Y-C et al. *JAMA Network Open.*
