# Supplementary material for: Discontinuation of Cholinesterase Inhibitors Following Initiation of Memantine and Admission to Long-Term Care Among Older Adults
Source: JAMA Netw Open. 2024 Nov 19;7(11):e2445878. doi: 10.1001/jamanetworkopen.2024.45878 (PMC11577138; doi:10.1001/jamanetworkopen.2024.45878)
Supplement: Supplement 2. — Data Sharing Statement [file jamanetwopen-e2445878-s002.pdf]

Data Sharing Statement

Lee. Discontinuation of Cholinesterase Inhibitors Following Initiation of Memantine and Admission to Long-Term Care Among Older Adults. *JAMA Netw Open*. Published November 19, 2024. doi:10.1001/jamanetworkopen.2024.45878

Data
Data available: No

Additional Information
Explanation for why data not available: We are unable to share data due to CMS data privacy rules. Medicare claims data are available through data use agreement with CMS.
